# Suppression of the c-erbB-2 gene product decreases transformation abilities but not the proliferation and secretion of proteases of SK-OV-3 ovarian cancer cells

**DOI:** 10.1038/sj.bjc.6690765

**Published:** 1999-11

**Authors:** K Wiechen, S Karaaslan, A Turzynski, M Dietel

**Affiliations:** Institute of Pathology, Universitätsklinikum Charité, Medizinische Fakultät der Humboldt-Universität Berlin, Schumannstr. 20/21, 10117 Berlin, Germany

**Keywords:** c-erbB-2 oncogene, ovarian cancer, single-chain antibody, proteases, cell transformation

## Abstract

The overexpression of the c-erbB-2 oncogene product has been reported in approximately 20–30% of human ovarian cancers and has been correlated with a poor prognosis in ovarian cancer patients. To investigate the function of p185^c-erbB-2^ in human ovarian cancer cells, a c-erbB-2-specific single-chain antibody (scFv-5R) was expressed in the c-erbB-2-overexpressing SK-OV-3 cell line using a retroviral expression vector. Eight individual clones expressing the single-chain antibody were isolated. These clones have a prominent retention of the cell surface p185^c-erbB-2^. In this study we compared the proliferation rate, the anchorage-independent growth, the secretion of matrix metalloproteases and of the urokinase-type plasminogen activator. The clones expressing the c-erbB-2 single-chain antibody, the control cells harbouring the empty vector and the parental SK-OV-3 cells they all had similar proliferation rates in the presence of 10% serum and secreted similar amounts of matrix metalloproteases and of the urokinase-type plasminogen activator. However, the expression of the c-erbB-2 oncogene product offers a strong growth advantage under serum-reduced conditions with 1% serum. In contrast to the parental SK-OV-3 and empty vector control cells, the scFv-5R-expressing clones were not able to grow anchorage-independently. These findings suggest that c-erbB-2 enhances transformation abilities of SK-OV-3 ovarian cancer cells without affecting the secretion of proteases and the proliferation of SK-OV-3 ovarian cancer cells in the presence of high concentrations of serum. © 1999 Cancer Research Campaign


*British Journal of Cancer* (1999) **81**(5), 790–795
© 1999 Cancer Research Campaign
Article no. bjoc.1999.0765

### Suppression of the c-erbB-2 gene product decreases transformation abilities but not the proliferation and secretion of proteases of SK-OV-3 ovarian cancer cells

**K Wiechen, S Karaaslan, A Turzynski and M Dietel**

Institute of Pathology, Universitätsklinikum Charité, Medizinische Fakultät der Humboldt-Universität Berlin, Schumannstr. 20/21, 10117 Berlin, Germany

**Summary** The overexpression of the c-erbB-2 oncogene product has been reported in approximately 20–30% of human ovarian cancers and has been correlated with a poor prognosis in ovarian cancer patients. To investigate the function of $p185^{c\text{-}erbB\text{-}2}$ in human ovarian cancer cells, a c-erbB-2-specific single-chain antibody (scFv-5R) was expressed in the c-erbB-2-overexpressing SK-OV-3 cell line using a retroviral expression vector. Eight individual clones expressing the single-chain antibody were isolated. These clones have a prominent retention of the cell surface $p185^{c\text{-}erbB\text{-}2}$. In this study we compared the proliferation rate, the anchorage-independent growth, the secretion of matrix metalloproteases and of the urokinase-type plasminogen activator. The clones expressing the c-erbB-2 single-chain antibody, the control cells harbouring the empty vector and the parental SK-OV-3 cells they all had similar proliferation rates in the presence of 10% serum and secreted similar amounts of matrix metalloproteases and of the urokinase-type plasminogen activator. However, the expression of the c-erbB-2 oncogene product offers a strong growth advantage under serum-reduced conditions with 1% serum. In contrast to the parental SK-OV-3 and empty vector control cells, the scFv-5R-expressing clones were not able to grow anchorage-independently. These findings suggest that c-erbB-2 enhances transformation abilities of SK-OV-3 ovarian cancer cells without affecting the secretion of proteases and the proliferation of SK-OV-3 ovarian cancer cells in the presence of high concentrations of serum. © 1999 Cancer Research Campaign

**Keywords:** c-erbB-2 oncogene; ovarian cancer; single-chain antibody; proteases; cell transformation

The amplification and/or overexpression of the c-erbB-2 (HER2/neu) oncogene product ($p185^{c\text{-}erbB\text{-}2}$) is a frequent event in different types of human cancer, including 20–30% of ovarian cancers. In ovarian cancer the overexpression of $p185^{c\text{-}erbB\text{-}2}$) characterizes a subgroup of patients with unfavourable tumour biology and poor prognosis (Berchuck et al, 1990; Meden et al, 1994). The c-erbB-2 gene encodes a 185 000 wild-type receptor tyrosine kinase with extensive homology to the epidermal growth factor receptor and the c-erbB-3 and c-erbB-4 gene products (Yamamoto et al, 1986; Kraus et al, 1989; Plowman et al, 1993). The members of the erbB family are co-expressed in various combinations in normal and neoplastic cells. Experimental evidence exists that the binding of particular ligands to the other receptors of the erbB family leads to the formation of heterodimers with $p185^{c\text{-}erbB\text{-}2}$ and that this tyrosine kinase receptor has an important function in modulating signal transduction, called lateral signal transduction (Graus-Porta et al, 1997).

It was previously demonstrated that the overexpression of the human c-erbB-2 oncogene is able to transform mouse NIH 3T3 fibroblasts and that the transformed fibroblasts are able to form tumours in mice (Hudziak et al, 1987). Furthermore in NIH 3T3 cells the point mutation-activated rat neu gene, the c-erbB-2 homologue, enhanced several metastasis/invasion-associated properties like cell growth, DNA synthesis rate, increased anchorage-independent growth rate and secretion of matrix-degrading enzymes (Yu and Hung, 1991). However, the function of the c-erbB-2 protooncogene and especially of the mutation-activated neu oncogene in rodent mesenchymal cells cannot be simply extrapolated to human ovarian cancer cells. The transition of normal epithelial cells to invasive carcinoma is a complex and multistage process involving enhanced proliferation, anchorage-independent growth, ability to degrade basal membrane components and cell migration. Therefore, systematic studies using ovarian cancer cells are necessary to determine whether the c-erbB-2 gene contributes to these parameters in ovarian cancer.

In this study, we investigated the role of the c-erbB-2 oncogene product in human ovarian cancer cells by the intracellular retention of $p185^{c\text{-}erbB\text{-}2}$ in c-erbB-2-overexpressing SK-OV-3 cells using an intracellular single-chain antibody (Graus-Porta et al, 1995). In addition to anchorage-dependent and anchorage-independent growth abilities we measured the secretion of matrix metalloproteases (MMPs) and of the urokinase-type plasminogen activator (uPA), which are key proteases implicated in extracellular matrix proteolysis (Chambers et al, 1995; Tamakoshi, et al, 1995). Our results demonstrate that the intracellular retention of $p185^{c\text{-}erbB\text{-}2}$ in SK-OV-3 ovarian cancer cells results in a loss of transformation abilities. However, in SK-OV-3 ovarian cancer cells the c-erbB-2 oncogene product is not involved in the secretion of MMPs, uPA and in cell proliferation in the presence of high concentrations of serum.

*Received 18 January 1999*
*Revised 25 March 1999*
*Accepted 12 March 1999*

*Correspondence to:* K Wiechen

### MATERIALS AND METHODS

### Materials and chemicals

The human ovarian cancer cell line SK-OV-3 was obtained from the American Type Culture Collection (HTB #77). The c-erbB-2-specific single-chain antibody scFv-5R vector pBP-5R was gratiously provided by NE Hynes (Friedrisch Miescher Institut, Basel, Switzerland). The scFv-5R coding sequence was excised from pBP-5R with *Eco*RI and *Sal*I, blunted with Klenow enzyme, ligated with *Bam*HI adaptors and cloned into the corresponding site of the bicistronic retroviral expression vector pLIXN (Clontech, Palo Alto, CA, USA), yielding pL5R. The proper orientation of the scFv-5R insert was proved by restriction analysis and by dideoxy sequencing. Phoenix-ampho retroviral packaging cells were kindly supplied by GP Nolan (Stanford University School of Medicine, CA, USA).

### Cell culture

Stock cultures of SK-OV-3 and Phoenix-ampho cells were maintained at 37°C in a humidified atmosphere of 5% carbon dioxide and 95% air in Dulbecco's modified Eagle's medium (DMEM) containing 10% fetal calf serum (FCS) and 2-mM glutamine.

### Expression of the c-erbB-2-directed scFv-5R in SK-OV-3 cells

A total of $2 \times 10^6$ Phoenix-ampho packaging cells were plated on 60-mm cell culture dishes. After 24 h, 4 $\mu$g of Quiagen-purified pL5R or pLIXN plasmid DNA were transfected as previously described (Pear et al, 1993). Two days after transfection, the virus-containing supernatant was harvested and used immediately for infection or stored frozen at –80°C. For the infection $2 \times 10^5$ SK-OV-3 cells were plated on 60-mm cell culture dishes. After 24 h infection was performed with the scFv-virus or neo control virus-containing medium in the presence of 8 $\mu$g ml$^{-1}$ polybrene (Sigma, St Louis, MO, USA). After 2 days the infected SK-OV-3 cells were subjected to selection in 1.5 mg ml$^{-1}$ geneticin (G418, Calbiochem, San Diego, CA, USA). Two weeks later individual G418-resistant clones were isolated and maintained with 0.75 mg ml$^{-1}$ G418. Pools of neo-transfected cells were used as a control.

### RNA isolation and Northern blot analysis

Total RNA was extracted by the acid guanidinium thiocyanate extraction method. Ten micrograms of total RNA per lane was size-fractionated on 0.8% agarose, 2.2% formaldehyde gels and blotted to nylon membranes by capillary transfer. The blots were hybridized with the *Eco*RI–*Sal*I scFv-5R cDNA fragment from pBP-5R and labelled with $^{32}$P-dCTP to a specific activity of $2 \times 10^8$ to $6 \times 10^8$ cpm per $\mu$g of DNA. After hybridization the blots were washed under high stringency conditions and exposed for 24 h at –80°C to Amersham MP film using intensifying screens. Equivalent loading of RNA was confirmed by ethidium bromide staining of the 28S and 18S rRNAs and by hybridizing with a glyceraldehyde-3-phosphate dehydrogenase (GAPDH) cDNA probe.

### Flow cytometry

A total of $2 \times 10^5$ SK-OV-3 cells were seeded on 60-mm culture dishes and after 24 h detached with 2 mM ethylenediamine tetraacetic acid (EDTA) in phosphate-buffered saline (PBS). The cells were then incubated with the p185$^{c\text{-}erbB\text{-}2}$-specific monoclonal antibody TA-1 (Oncogene Science) at 0.2 $\mu$g ml$^{-1}$. Cells were incubated on ice for 1 h and subsequently incubated for 45 min with a R-phycoerythrin (PE)-labelled goat anti-mouse conjugate (Sigma, St Louis, MO, USA). The cells were then analysed with a Becton-Dickinson flow cytometer using the 488 nm excitation line of the argon laser. PE fluorescence was collected through a 585 nm band pass filter. Mouse IgG with irrelevant specificity was used as a control.

### Monolayer cell growth

The growth rates of SK-OV-3 cells and their derivatives were assessed by measuring relative increases in cell number using an XTT assay (Boehringer Mannheim, Germany). One thousand cells were plated in 96-well flat-bottom culture wells in 0.1 ml of culture medium supplemented with 10% or 1% FCS. From day 1 to day 6 XTT reagent was added to one row (8 wells) of the plate as recommended by the manufacturer. Formazan dye was measured in a microplate reader after 4 h at 470 nm. The proliferation experiments were performed a least three times independently.

### Anchorage-independent cell growth

Anchorage-independent cell growth was measured in microtitre plates coated with poly(2-hydroxyethyl methacrylate) (poly(HEMA)) as described previously (Fukazawa et al, 1995). Briefly, 50 $\mu$l of poly(HEMA) (Sigma), dissolved at 5 mg ml$^{-1}$ in 95% ethanol was applied into wells of 96-well plates. The plates were allowed to dry for 2 days at 37°C. Cells were seeded in a volume of 150 $\mu$l into each well at a density of 1000 cells per well. On the following days 1–6 50 $\mu$l of XTT reagent (Boehringer Mannheim, Germany) was added and further incubated for 4 h. The absorbance was measured at 470 nm.

### Measurement of proteases

Secreted proteases were measured in serum-free conditioned cell culture medium. Then, $2 \times 10^5$ cells were seeded on 12-well tissue culture plates in DMEM supplemented with 10% FCS and cultured overnight. The cells were washed two times with serum-free DMEM with 0.1% bovine serum albumin (BSA) and incubated with 250 $\mu$l DMEM/0.1% BSA for 16 h. Matrix metalloproteases were determined using a zymographic assay. Briefly, the culture supernatant was subjected to 10% sodium dodecyl sulphate polyacrylamide gel electrophoresis (SDS-PAGE) with 1.5% gelatin incorporated in the separating gel under non-reducing conditions. The gel was then washed with 2.5% Triton X-100 in 50 mM Tris–HCl, pH 7.5 and incubated for 16 h in 0.15 M sodium chloride, 10 mM calcium chloride in 50 mM Tris–HCl, pH 7.5. Afterwards the gel was stained with Coomassie brilliant blue and destained. uPA concentrations were determined by using a commercially available enzyme-linked immunosorbent assay (ELISA) kit (American Diagnostica, Greenwich, CT, USA) as recommended by the manufacturer.

© 1999 Cancer Research Campaign

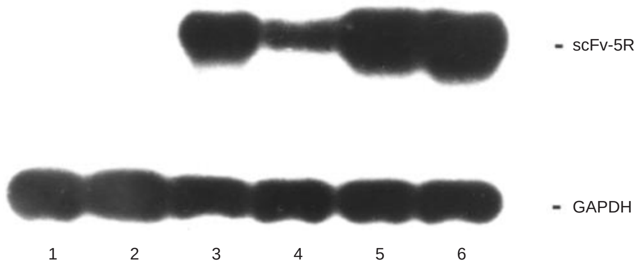

**Figure 1** (Upper panel) Expression of scFv-5R RNA. Ten micrograms of total RNA per lane was electrophoresed, blotted and probed with a scFv-5R-specific $^{32}$P-labelled probe. Lane 1, SK-OV-3, lane 2, neo control cells, lanes 3–6, representative individual cell clones expressing scFv-5R. (Lower panel) Hybridization with a glyceraldehyde-3-phosphate dehydrogenase (GAPDH) cDNA probe to confirm equal loading amounts

### RESULTS

We expressed the c-erbB-2-specific single-chain antibody scFv-5R of Hynes and collaborators (Graus-Porta et al, 1995) in the c-erbB-2-overexpressing SK-OV-3 ovarian cancer cell line. This single-chain antibody has been shown to interfere with the function of p185$^{c\text{-}erbB\text{-}2}$ in human cancer cells of different histologic origin (Beerli et al, 1995). Eight stable individual clones were isolated that express high levels of the scFv-5R RNA as revealed by Northern blot hybridization (Figure 1). These clones have an almost complete retention of the cell surface p185$^{c\text{-}erbB\text{-}2}$ level as measured by flow cytometric analysis (Figure 2). The clones were morphologically similar to the parental SK-OV-3 cell line and to the control cells carrying the empty vector with the neo selectable marker (not shown).

The influence of cell surface p185$^{c\text{-}erbB\text{-}2}$ on cell proliferation was measured by comparing relative increases in cell number with a colourimetric XTT assay. In these experiments the growth rates of the parental SK-OV-3 cells, the neo control cells expressing the empty vector and the individual clones expressing scFv-5R did not show significant differences in the presence of 10% FCS (Figure 3A). However, the anchorage-dependent cell proliferation of the individual clones expressing scFv-5R was strongly inhibited in the presence of 1% FCS (Figure 3B).

We then compared the growth rates of the cells in microtitre plates coated with poly(HEMA). Under these conditions the cells were not able to adhere to the bottom of the wells and to spread. The parental SK-OV-3 cells and the neo control cells show rapid growth under anchorage-independent conditions and formed three-dimensional colonies in the coated wells. In contrast, the cell clones expressing scFv-5R were not able at all to grow under anchorage-independent conditions (Figure 3C). We have in addition performed experiments where the cells were immersed in soft agar with comparable results (not shown).

Secretion of MMPs was measured using a zymographic assay (Figure 4A). In conditioned cell culture medium of SK-OV-3 cells the 72 kDa MMP-2 (gelatinase A, MMP-2) is easily detectable. The 92 kDa MMP (gelatinase B, MMP-9) is not detectable in the conditioned medium. No significant alterations in MMP-2 levels are seen in the clones expressing scFv-5R or the control cells as compared to the parental SK-OV-3 cells.

In addition, we have measured the amounts of uPA in the conditioned medium of SK-OV-3 cells and their derivatives using an

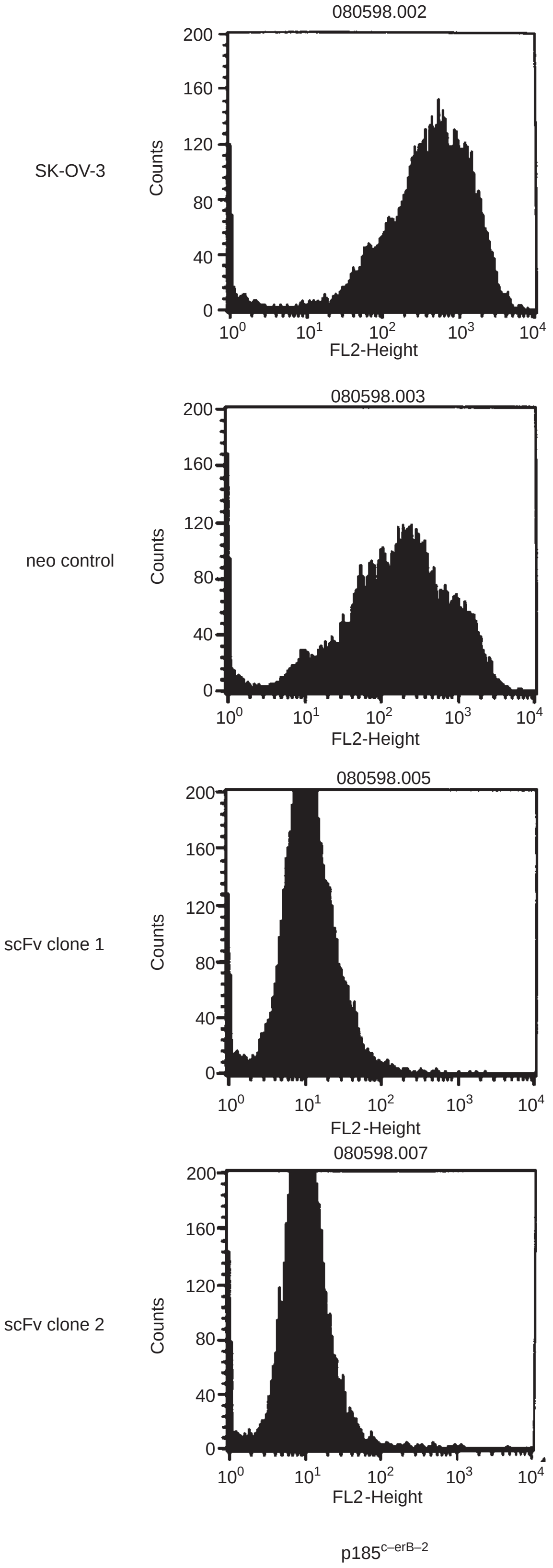

**Figure 2** Detection of p185$^{c\text{-}erbB\text{-}2}$ by a flow cytometric assay. The cells were detached with EDTA/PBS and subsequently incubated with a c-erbB-2-specific monoclonal antibody and then with a R-phycoerythrin (PE)-labelled secondary antibody. Loss of cell surface p185$^{c\text{-}erbB\text{-}2}$ in cell clones expressing the scFv-5R. Data shown are from 2 representative individual cell clones

*© 1999 Cancer Research Campaign*

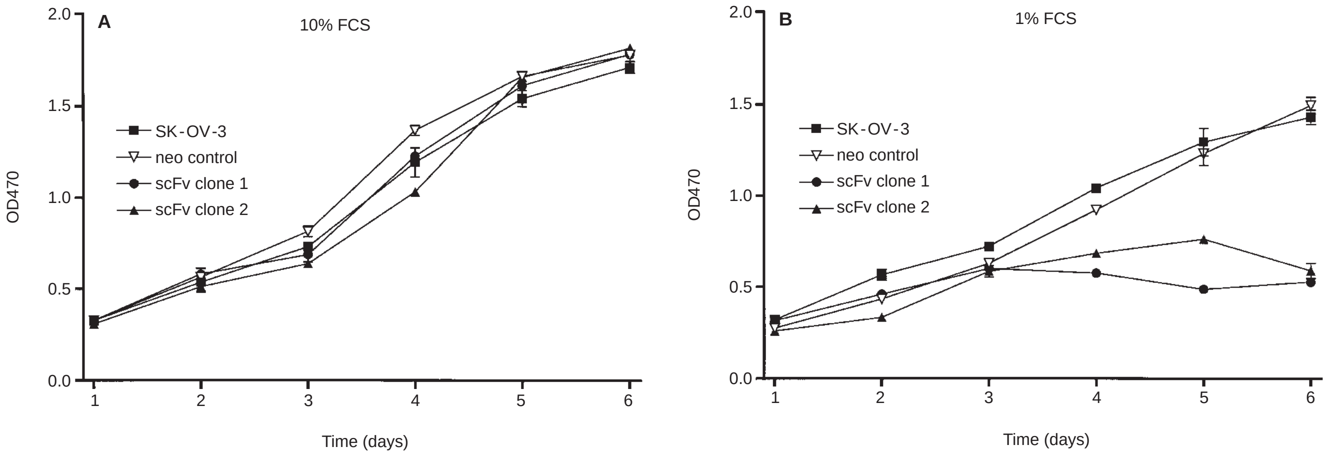

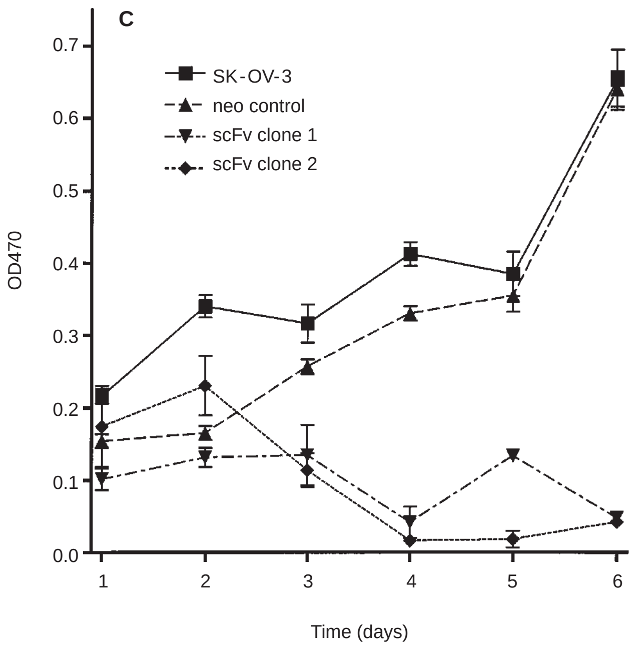

**Figure 3** (**A** and **B**) Similar growth rates among the scFv-5R-expressing clones and the control cells in the presence of 10% FCS (**A**) and growth inhibition of the scFv-expressing clones in the presence of 1% FCS (**B**). Cells ($1 \times 10^3$) were plated on microtitre plates. Relative increases in cell number were measured using an XTT test on different days for 6 days. The OD 470 is given as mean ± s.e.m. (**C**) Loss of anchorage-independent growth abilities of cell clones expressing scFv-5R. One thousand cells were plated on poly(HEMA) coated microtitre plates. Relative increases in cell number were measured using a XTT test on different days for 6 days. The OD 470 is given as mean ± s.e.m. Data shown are from parental SK-OV-3 cells, neo control cells and two representative cell clones expressing scFv-5R

ELISA assay. As shown in Figure 4B similar concentrations of uPA are present in the conditioned medium of SK-OV-3 cells, the neo control cells and the clones. The small differences of the uPA concentrations between the individual cell clones may represent a clonal variation. This is reflected by the very similar uPA values when comparing the data of the controls (SK-OV-3 and neo) with the sum data from the individual clones (Figure 4B, bar on the right)

### DISCUSSION

Clinical data suggest an important function of $p185^{c\text{-}erbB\text{-}2}$ in malignant epithelial tumours of different histologic origin including ovarian cancer. Although c-erbB-2 overexpression was previously shown to be correlated with a poor prognosis (Berchuck et al, 1990; Meden et al, 1994) the precise functions of $p185^{c\text{-}erbB\text{-}2}$ in ovarian cancer are not very well understood. The majority of data about the function of c-erbB-2 was derived from experiments using overexpression of the c-erbB-2 gene in rodent mesenchymal cells. To investigate the role of $p185^{c\text{-}erbB\text{-}2}$ in human ovarian cancer cells, we have used the c-erbB-2-specific intracellular single-chain antibody of Hynes and collaborators (Graus-Porta et al, 1995) to suppress the c-erbB-2 gene product in the overexpressing SK-OV-3 cell line. This single-chain antibody causes specific intracellular retention of the c-erbB-2 gene product. It does not interfere with the synthesis or stability of $p185^{c\text{-}erbB\text{-}2}$. However, in tumour cells expressing scFv-5R the c-erbB-2 gene product shows a faster electrophoretic mobility which may be due to hypoglycosylation by intracellular retention (Graus-Porta et al, 1995). It was, in addition, previously shown that the expression of the EGF receptor and the c-erbB-3 gene product are not influenced by the scFv-5R single-chain antibody (Graus-Porta et al, 1995).

Individual cell clones with a complete retention of the cell surface $p185^{c\text{-}erbB\text{-}2}$ lost the ability of the parental SK-OV-3 tumour cells to grow anchorage-independently. The ability of the c-erbB-2 oncogene to transform cells and to induce anchorage-independent growth abilities was first described in NIH 3T3 mouse fibroblasts

*© 1999 Cancer Research Campaign*

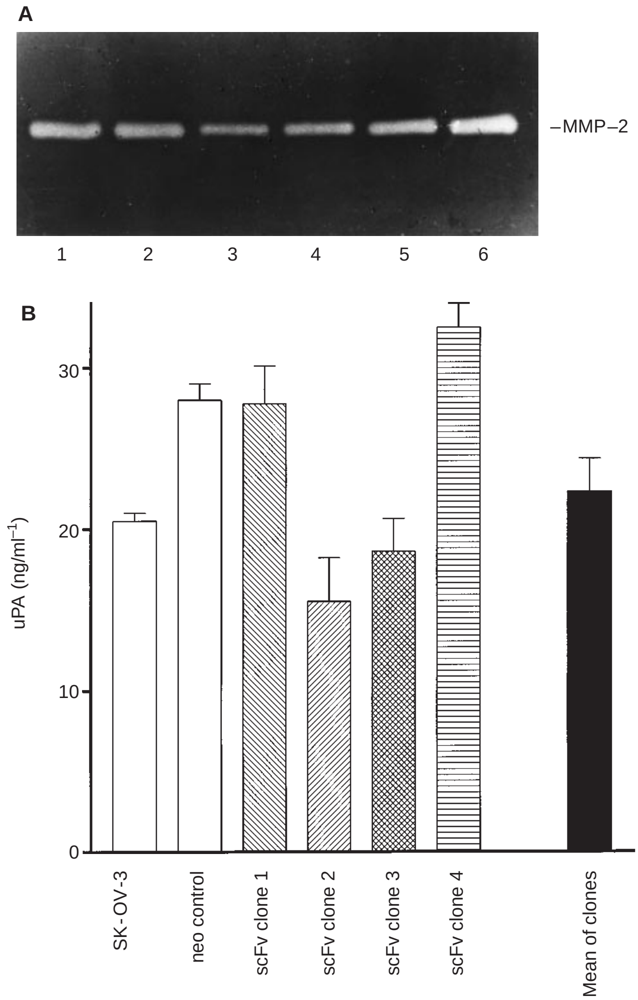

**Figure 4** (**A**) Secretion of similar amounts of matrix metalloproteases by SK-OV-3 cells (lane 1), neo control cells (lane 2) and 4 representative scFv-5R-expressing cell clones (lanes 3–6). A total of $2 \times 10^5$ cells were seeded on 12-well tissue culture plates. The cells were incubated with DMEM/0.1% BSA for 16 h. Cultured media were subjected to SDS-PAGE (gel containing 1.5% gelatin) without reducing agent. The gel was afterwards incubated in reaction buffer and stained with Coomassie Brilliant Blue, and destained. The position of the 72-kDa matrix metalloprotease (MMP-2) is indicated. (**B**) Secretion of similar amounts of uPA by SK-OV-3 cells, neo control cells and 4 representative scFv-5R-expressing cell clones. The conditioned medium was prepared as described above. The uPA concentration was measured using a commercially available ELISA assay. Left-hand bars, uPA concentration of SK-OV-3, neo controls and 4 representative individual scFv-expressing cell clones (*Y*-axis in ng ml$^{-1}$ ± s.e.m.). Right-hand bar, sums of uPA concentrations of the 4 scFv-5R-expressing cell clones

(Hudziak et al, 1987). However, in SV40-immortalized human bronchial epithelial cells the overexpression of c-erbB-2 was not sufficient to enhance anchorage-independent growth abilities and only one cell clone was able to form tumours in vivo (Noguchi et al., 1993). Other experiments using tumour cell lines also show different results when analysing the c-erbB-2 function in cell transformation. The enhanced expression of c-erbB-2 in SK-OV-3 cells after an animal passage resulted in enhanced anchorage-independent growth abilities (Yu et al, 1993). In contrast, in human MDA-MB-435 breast cancer cells that express only low amounts of endogenous p185$^{c\text{-}erbB\text{-}2}$ the overexpression of c-erbB-2 does not result in enhanced anchorage-independent growth abilities (Tan et al, 1997).

The intracellular retention of p185$^{c\text{-}erbB\text{-}2}$ does not influence the anchorage-dependent proliferation of SK-OV-3 ovarian cancer cells in the presence of high concentrations of serum. Recently it was shown that transfection of a human c-erbB-2 vector into hormone-dependent MCF-7 human breast cancer cells is able to inhibit cell growth and to induce cell differentiation (Giani et al, 1998). Although we have not analysed the sensitivity of SK-OV-3 to steroid hormones, the growth of the SK-OV-3 derivatives in the presence of 10% serum was independent from the cell surface p185$^{c\text{-}erbB\text{-}2}$.

However, the expression of the c-erbB-2 gene product offers a strong growth advantage under serum-reduced conditions. Therefore cell transformation consisting of enhanced growth abilities under serum-reduced or anchorage-independent conditions and cell proliferation in the presence of high concentrations of serum of SK-OV-3 ovarian cancer cells may be mediated by different non-overlapping signal transduction pathways.

Previously, Deshane and colleagues have used another single-chain antibody to suppress c-erbB-2 in cancer cells. This single-chain antibody was expressed transiently using the adenovirus-polylysine technique and a recombinant adenovirus (Deshane et al, 1995*a*, 1995*b*, 1996). They reported specific induction of apoptosis in SK-OV-3 and other c-erbB-2-overexpressing human cancer cell lines. The increased rate of apoptosis in these experiments resulted in a decrease of anchorage-dependent (Deshane et al, 1996) and anchorage-independent growth abilities (Deshane et al, 1995*a*). In addition the expression of this single-chain antibody using adenovirus transfer was able to inhibit tumour growth in vivo (Deshane et al, 1995*b*). In our experiments, the single-chain antibody scFv-5R was expressed stably for a long-term using a retroviral vector. Increased amounts of dead cells that may be attributed to apoptosis were not observed in our experiments with SK-OV-3 cells under anchorage-dependent conditions. The induction of apoptosis in SK-OV-3 cells under anchorage-dependent conditions observed by Deshane and colleagues may be due to the very high level of transient expression by the adenovirus vector and/or be directly caused by the different single-chain antibody targeted against the c-erbB-2 gene product.

In addition to cell proliferation and transformation, invasive and metastatic tumour growth involves the degradation of basal membrane components and of the surrounding stroma by proteases. Therefore we measured the secreted amounts of MMPs and uPA. The secretion of MMPs is independent of p185$^{c\text{-}erbB\text{-}2}$ in SK-OV-3 ovarian cancer cells. In contrast, in human MDA-MB-435 breast cancer cells, the overexpression of the c-erbB-2 oncogene enhances the intrinsic metastatic potential by increasing secreted activities of MMPs (Tan et al, 1997).

Another key protease implicated in invasive tumour growth, uPA, was previously shown to be significantly elevated in peritoneal metastases of ovarian carcinomas as compared to primary tumours (Schmalfeldt et al, 1995). In SK-OV-3 ovarian cancer cells the secreted amounts of uPA are independent of p185$^{c\text{-}erbB\text{-}2}$. In contrast in mouse NIH 3T3 fibroblasts and in human H460 lung cancer cells uPA is up-regulated by the expression of p185$^{c\text{-}erbB\text{-}2}$ (Gum et al, 1995). On the basis of our experiments we cannot exclude that other types of proteases are regulated p185$^{c\text{-}erbB\text{-}2}$-dependently in SK-OV-3 cells.

To sum up, parameters involved in tumour progression are regulated by the c-erbB-2 gene product in a cell type-specific fashion. Our results show that the c-erbB-2 gene product regulates the transformation abilities in SK-OV-3 ovarian cancer cells. In

 © 1999 Cancer Research Campaign addition we have previously shown that SK-OV-3 cells express EGF receptors together with $p185^{c\text{-}erbB\text{-}2}$ and that $p185^{c\text{-}erbB\text{-}2}$ is involved in EGF-induced tumour cell motility in SK-OV-3 cells (Weichen et al, submitted). Therefore it is likely that in SK-OV-3 cells $p185^{c\text{-}erbB\text{-}2}$ modulates signal transduction pathways to the cytoskeleton and the actomyosin system and/or transcriptionally regulates involved genes.

Ovarian cancer is the most lethal tumour of the female genital tract and continues to be the major cause of female cancer mortality, largely as a function of early abdominal seeding producing peritoneal carcinosis and ascites. In contrast to other human epithelial malignancies, the generation of distant metastases is uncommon in ovarian cancer. This may be reflected by the $p185^{c\text{-}erbB\text{-}2}$ independence of the secretion of matrix-degrading enzymes in the SK-OV-3 cell system. This point of view is supported by the fact that MMP levels are significantly higher in cervical and endometrial cancer that are characterized by metastatic progression compared to ovarian cancer (Tamakoshi et al, 1995). The reorganization of the cytoskeleton by the overexpression of $p185^{c\text{-}erbB\text{-}2}$ involved in enhanced anchorage-independent growth may contribute to the formation of malignant ascites and to the peritoneal spread of tumour cells characteristic for ovarian cancer and thus contribute to the poor prognosis of c-erbB-2-overexpressing tumours.

### ACKNOWLEDGEMENTS

We would like to thank Dr NE Hynes, Basel, Switzerland, for the gift of the c-erbB-2 single-chain antibody vector pBP-5R and Dr G P Nolan, Stanford, USA, for the Phoenix-ampho retroviral packaging cells. We thank M Grips for help with the poly(HEMA) assay, D Zacher and S Wurr for excellent technical assistance, and G Stemmler for editorial help. This work was supported by grant 10-0968-Wi 1 from Deutsche Krebshilfe to KW and MD.

### REFERENCES

Beerli RR, Graus-Porta D, Woods-Cook K, Chen X, Yarden Y and Hynes NE (1995) Neu differentiation factor activation of ErbB-3 and ErbB-4 is cell specific and displays a differential requirement for ErbB-2. *Mol Cell Biol* **15**: 6496–6505

Berchuck A, Kamel A, Whitaker R, Kerns B, Olt G, Kinney R, Soper JT, Dodge R, Clarke-Pearson DL, Marks P, McKenzie S, Yin S and Bast RC Jr (1990) Overexpression of her-2/neu is associated with poor survival in advanced epithelial ovarian cancer. *Cancer Res* **50**: 4087–4091

Chambers SK, Gertz RE, Ivins CM and Kacinski BM (1995) The significance of urokinase-type plasminogen activator, its inhibitors, and its receptor in ascites of patients with epithelial ovarian cancer. *Cancer* **75**: 1627–1633

Deshane J, Cabrera G, Grim J, Siegal GP, Pike J, Alvarez RD and Curiel DT (1995) Targeted eradication of ovarian cancer mediated by intracellular expression of anti-erbB-2 single-chain antibody. *Gynecol Oncol* **59**: 8–14

Deshane J, Grim J, Loechel S, Siegal GP, Alvarez RD and Curiel DT (1996) Intracellular antibody against erbB-2 mediates targeted tumor cell eradication by apoptosis. *Cancer Gene Ther* **3**: 89–98

Deshane J, Siegal GP, Alvarez RD, Wang MH, Feng M, Cabrera G, Liu T, Kay M and Curiel DT (1995) Targeted tumor killing via an intracellular antibody against erbB-2. *J Clin Invest* **96**: 2989–2989

Fukazawa H, Mizuno S and Uehara Y (1995) A microplate assay for quantitation of anchorage-independent growth of transformed cells. *Anal Biochem* **228**: 83–90

Giani C, Casalini P, Pupa SM, De Vecchi R, Ardini E, Colnaghi MI, Giordano A and Menard S (1998) Increased expression of c-erbB-2 in hormone-dependent breast cancer cells inhibits cell growth and induces differentiation. *Oncogene* **17**: 425–432

Graus-Porta D, Beerli RR and Hynes NE (1995) Single-chain antibody-mediated intracellular retention of ErbB-2 impairs Neu differentiation factor and epidermal growth factor signaling. *Mol Cell Biol* **15**: 1182–1191

Graus-Porta D, Beerli RR, Daly JM and Hynes NE (1997) ErbB-2, the preferred heterodimerization partner of all ErbB receptors, is a mediator of lateral signaling. *EMBO J* **16**: 1647–1655

Gum R, Wang SW, Lengyel E, Yu D, Hung M-C, Juarez J and Boyd D (1995) Up-regulation of urokinase-type plasminogen activator by expression by the HER2/neu proto-oncogene. *Anticancer Res* **15**: 1167–1172

Hudziak RM, Schlessinger J and Ullrich A (1987) Increased expression of the putative growth factor receptor $p185^{HER2}$ causes transformation and tumorigenesis of NIH 3T3 cells. *Proc Natl Acad Sci USA* **84**: 7159–7163

Kraus MH, Issing W, Miki T, Popescu NC and Aaronson SA (1989) Isolation and characterization of ERBB3, a third member of the ERBB/epidermal growth factor receptor family: evidence for overexpression in a subset of human mammary tumors. *Proc Natl Acad Sci USA* **86**: 9193–9197

Meden H, Marx D, Rath W, Kron M, Fattahi-Meibodi A, Hinney B, Kuhn W and Schauer A (1994) Overexpression of the oncogene c-erbB2 in primary ovarian cancer: evaluation of the prognostic value in a Cox proportional hazards multiple regression. *Int J Gynecol Pathol* **13**: 45–53

Noguchi M, Murakami M, Bennett W, Lupu R, Hui F, Harris CC and Gerwin BL (1993) Biological consequences of overexpression of a transfected c-erbB-2 gene in immortalized human bronchial epithelial cells. *Cancer Res* **53**: 2035–2043

Pear WS, Nolan GP, Scott ML and Baltimore D (1993) Production of high-titer helper-free retroviruses by transient transfection. *Proc Natl Acad Sci USA* **90**: 8392–8396

Plowman GD, Culouscu J-M, Whitney GS, Green JM, Carlton GW, Foy L, Neubauer MG and Shoyab M (1993) Ligand-specific activation of HER4/p180erbB4, a fourth member of the epidermal growth factor receptor family. *Proc Natl Acad Sci USA* **90**: 1746–1750

Schmalfeldt B, Kuhn W, Reuning U, Pache L, Dettmar P, Schmitt M, Jänicke F, Höfler H and Graeff H (1995) Primary tumor and metastasis in ovarian cancer differ in their content of urokinase-type plasminogen activator, its receptor, and inhibitors types 1 and 2. *Cancer Res* **55**: 3958–3963

Tamakoshi K, Kikkawa F, Nawa A, Ishikawa H, Mizuno K, Tamakoshi A, Yamagata S, Suganuma N and Tomoda Y (1995) Characterization of extracellular matrix-degrading proteinase and its inhibitor in gynecologic cancer tissues with clinically different metastatic form. *Cancer* **76**: 2565–2571

Tan M, Yao J and Yu D (1997) Overexpression of the c-erbB-2 gene enhanced intrinsic metastasis potential in human breast cancer cells without increasing their transformation abilities. *Cancer Res* **57**: 1199–1205

Yamamoto T, Ikawa S, Akiyama T, Semba K, Nomura N, Miyajima N, Saito T and Toyoshima K (1986) Similarity of protein encoded by the human c-erb-B-2 gene to epidermal growth factor receptor. *Nature (London)* **319**: 230–234

Yu DH and Hung M-C (1991) Expression of activated rat neu oncogene is sufficient to induce experimental metastasis in 3T3 cells. *Oncogene* **6**: 1991–1996

Yu D, Wolf JK, Scanlon M, Price JE and Hung M-C (1993) Enhanced c-erbB-2/neu expression in human ovarian cancer cells correlates with more severe malignancy that can be suppressed by E1A. *Cancer Res* **53**: 891–898

© 1999 Cancer Research Campaign

## References

[PDF_00551] Beerli R. R., Graus-Porta D., Woods-Cook K., Chen X., Yarden Y., Hynes N. E. (1995). Neu differentiation factor activation of ErbB-3 and ErbB-4 is cell specific and displays a differential requirement for ErbB-2.. Mol Cell Biol.

[PDF_00554] Berchuck A., Kamel A., Whitaker R., Kerns B., Olt G., Kinney R., Soper J. T., Dodge R., Clarke-Pearson D. L., Marks P. (1990). Overexpression of HER-2/neu is associated with poor survival in advanced epithelial ovarian cancer.. Cancer Res.

[PDF_00558] Chambers S. K., Gertz R. E., Ivins C. M., Kacinski B. M. (1995). The significance of urokinase- type plasminogen activator, its inhibitors, and its receptor in ascites of patients with epithelial ovarian cancer.. Cancer.

[PDF_00561] Deshane J., Cabrera G., Grim J. E., Siegal G. P., Pike J., Alvarez R. D., Curiel D. T. (1995). Targeted eradication of ovarian cancer mediated by intracellular expression of anti-erbB-2 single-chain antibody.. Gynecol Oncol.

[PDF_00564] Deshane J., Grim J., Loechel S., Siegal G. P., Alvarez R. D., Curiel D. T. (1996). Intracellular antibody against erbB-2 mediates targeted tumor cell eradication by apoptosis.. Cancer Gene Ther.

[PDF_00567] Deshane J., Siegal G. P., Alvarez R. D., Wang M. H., Feng M., Cabrera G., Liu T., Kay M., Curiel D. T. (1995). Targeted tumor killing via an intracellular antibody against erbB-2.. J Clin Invest.

[PDF_00570] Fukazawa H., Mizuno S., Uehara Y. (1995). A microplate assay for quantitation of anchorage-independent growth of transformed cells.. Anal Biochem.

[PDF_00572] Giani C., Casalini P., Pupa S. M., De Vecchi R., Ardini E., Colnaghi M. I., Giordano A., Ménard S. (1998). Increased expression of c-erbB-2 in hormone-dependent breast cancer cells inhibits cell growth and induces differentiation.. Oncogene.

[PDF_00579] Graus-Porta D., Beerli R. R., Daly J. M., Hynes N. E. (1997). ErbB-2, the preferred heterodimerization partner of all ErbB receptors, is a mediator of lateral signaling.. EMBO J.

[PDF_00576] Graus-Porta D., Beerli R. R., Hynes N. E. (1995). Single-chain antibody-mediated intracellular retention of ErbB-2 impairs Neu differentiation factor and epidermal growth factor signaling.. Mol Cell Biol.

[PDF_00582] Gum R., Wang S. W., Lengyel E., Yu D., Hung M. C., Juarez J., Boyd D. (1995). Up-regulation of urokinase-type plasminogen activator expression by the HER2/neu proto-oncogene.. Anticancer Res.

[PDF_00585] Hudziak R. M., Schlessinger J., Ullrich A. (1987). Increased expression of the putative growth factor receptor p185HER2 causes transformation and tumorigenesis of NIH 3T3 cells.. Proc Natl Acad Sci U S A.

[PDF_00589] Kraus M. H., Issing W., Miki T., Popescu N. C., Aaronson S. A. (1989). Isolation and characterization of ERBB3, a third member of the ERBB/epidermal growth factor receptor family: evidence for overexpression in a subset of human mammary tumors.. Proc Natl Acad Sci U S A.

[PDF_00593] Meden H., Marx D., Rath W., Kron M., Fattahi-Meibodi A., Hinney B., Kuhn W., Schauer A. (1994). Overexpression of the oncogene c-erb B2 in primary ovarian cancer: evaluation of the prognostic value in a Cox proportional hazards multiple regression.. Int J Gynecol Pathol.

[PDF_00597] Noguchi M., Murakami M., Bennett W., Lupu R., Hui F., Harris C. C., Gerwin B. I. (1993). Biological consequences of overexpression of a transfected c-erbB-2 gene in immortalized human bronchial epithelial cells.. Cancer Res.

[PDF_00601] Pear W. S., Nolan G. P., Scott M. L., Baltimore D. (1993). Production of high-titer helper-free retroviruses by transient transfection.. Proc Natl Acad Sci U S A.

[PDF_00604] Plowman G. D., Culouscou J. M., Whitney G. S., Green J. M., Carlton G. W., Foy L., Neubauer M. G., Shoyab M. (1993). Ligand-specific activation of HER4/p180erbB4, a fourth member of the epidermal growth factor receptor family.. Proc Natl Acad Sci U S A.

[PDF_00608] Schmalfeldt B., Kuhn W., Reuning U., Pache L., Dettmar P., Schmitt M., Jänicke F., Höfler H., Graeff H. (1995). Primary tumor and metastasis in ovarian cancer differ in their content of urokinase-type plasminogen activator, its receptor, and inhibitors types 1 and 2.. Cancer Res.

[PDF_00612] Tamakoshi K., Kikkawa F., Nawa A., Ishikawa H., Mizuno K., Tamakoshi A., Yamagata S., Suganuma N., Tomoda Y. (1995). Characterization of extracellular matrix-degrading proteinase and its inhibitor in gynecologic cancer tissues with clinically different metastatic form.. Cancer.

[PDF_00616] Tan M., Yao J., Yu D. (1997). Overexpression of the c-erbB-2 gene enhanced intrinsic metastasis potential in human breast cancer cells without increasing their transformation abilities.. Cancer Res.

[PDF_00619] Yamamoto T., Ikawa S., Akiyama T., Semba K., Nomura N., Miyajima N., Saito T., Toyoshima K. (1986). Similarity of protein encoded by the human c-erb-B-2 gene to epidermal growth factor receptor.. Nature.

[PDF_00622] Yu D. H., Hung M. C. (1991). Expression of activated rat neu oncogene is sufficient to induce experimental metastasis in 3T3 cells.. Oncogene.

[PDF_00624] Yu D., Wolf J. K., Scanlon M., Price J. E., Hung M. C. (1993). Enhanced c-erbB-2/neu expression in human ovarian cancer cells correlates with more severe malignancy that can be suppressed by E1A.. Cancer Res.

